# Pharmacological inhibition of MCL-1 disrupts mitochondrial cristae and depletes the human neural progenitor cell pool

**DOI:** 10.64898/2025.12.12.694056

**Published:** 2025-12-13

**Authors:** Marina R. Hanna, Madison Yarbrough, Melanie Gil, James Costanzo, Vivian Gama

**Affiliations:** 1 Vanderbilt University, Vanderbilt Brain Institute, Nashville, TN; 2 Vanderbilt University, Cell and Developmental Biology, Nashville, TN.; 3 Vanderbilt University, Vanderbilt Center for Stem Cell Biology, Nashville, TN

**Keywords:** neural stem cells, MCL-1, mitochondria, cristae, MICOS, OPA1, intermediate progenitors

## Abstract

Myeloid Cell Leukemia-1 (MCL-1) is an anti-apoptotic protein that is crucial for early neurodevelopment. Loss of MCL-1 results in embryonic lethal neurodevelopmental defects that cannot be rescued by other anti-apoptotic proteins of the BCL-2 family. Here, we find a non-apoptotic role for MCL-1 in sustaining mitochondrial cristae integrity, fatty acid oxidation (FAO), and neural progenitor identity in human neural stem cells (hNSCs). Pharmacological inhibition of MCL-1 with S63845 disrupts mitochondrial ultrastructure, leading to swollen mitochondria with disorganized cristae and destabilization of the OPA1-MICOS machinery that governs inner membrane architecture. These structural defects are accompanied by impaired lipid droplet accumulation and altered expression of β-oxidation enzymes, revealing a tight link between cristae architecture and metabolic competence. Functionally, in the absence of caspase-mediated cell death, MCL-1 inhibition selectively depletes intermediate progenitor cells and alters neuronal differentiation without affecting proliferation, indicating a direct role in lineage progression. Together, our findings identify MCL-1 as a modulator of cristae organization, linking lipid metabolism to neural progenitor fate. This work establishes mitochondrial inner membrane architecture as an instructive determinant of human neurogenesis and highlights the non-canonical MCL-1 functions as critical regulators of human brain development

## Introduction

Neurodevelopment relies on the precise coordination of cellular processes that determine the fate of multipotent *PAX6*-expressing radial glial cells (RGCs)^1–3^. RGCs are the major neural stem cells (NSCs) that give rise to glia and neurons in the human cortex^4,5^. The fate of these NSCs relies on their ability to undergo symmetric or asymmetric division, a process that ultimately determines whether they self-renew or differentiate into specialized cell types^4,5^. NSCs give rise to intermediate progenitor cells (IPCs)^6,7^, which are *EOMES* (TBR2)-expressing^8,9^ cortex-specific transit amplifying cells^10^ committed to producing glutamatergic projection neurons of layers two through six^11,12^. Given the high bioenergetic demands and metabolic complexity of NSCs, mitochondrial morphology and function disruption can impair critical stem cell transitions during neurogenesis^13–15^. Despite this, the precise role of mitochondrial morphology and function in these intricate transition states is poorly understood.

Myeloid cell leukemia-1 (MCL-1) is a mitochondrial membrane protein identified as a regulator of mitochondrial morphology through undefined mechanisms that are independent of its canonical anti-apoptotic function^16–20^. Initially only recognized as a member of the B-cell lymphoma 2 (BCL-2) family for its BCL-2 homology-3 (BH3) domain and role in preventing cell death at the outer mitochondrial membrane (OMM)^21^, MCL-1 has been well-established to have non-apoptotic functions^17,18,22–26^. Disruption of MCL-1 causes mitochondrial dysfunction in human cardiomyocytes^20,23^, as well as improper development of hematopoietic stem cells^27^, B cell and T cell progenitors^28^, activated B cells^29^, and the central nervous system^30^. MCL-1 has been found to also localize at the inner mitochondrial membrane (IMM) where it potentially associates with cristae shaping proteins, such as optic atrophy type 1 (OPA1)^18,19,23^. However, the function of MCL-1 at the IMM is not well understood.

Previous work has reported a close association between MCL-1, mitochondrial morphology, and pluripotent stem cell identity of human embryonic stem cells (hESCs)^17^. Knockdown (KD) of *MCL-1* in hESCs^17^ results in an elongated mitochondrial network, a phenotype that has been linked to differentiation^31^. Moreover, *MCL-1*^*KD*^ hESCs exhibited a loss of key pluripotent transcription factors and identity markers, *OCT4* and *NANOG*^32,33^. Other studies have established a postmitotic period of fate plasticity controlled by mitochondrial morphology during mouse brain development^34,35^. When mitochondrial fusion is disrupted, NSC divide symmetrically, thus expanding the pool of progenitor cells. Conversely, disruption of mitochondrial fragmentation drives NSCs towards premature exit from multipotency, thus inducing lineage differentiation and depletion of the progenitor pool^34,35^. However, the role of MCL-1 in regulating human neurogenesis has not been fully explored.

*In vivo* mouse studies highlight the importance of MCL-1 in mouse embryonic survival and proper fetal neurodevelopment^26^. A gene swap approach in which the coding sequence of the *Mcl-1* gene in mice was replaced with other anti-apoptotic genes *BCL-2* (*Mcl-1*^*BCL−2*^), *BCL-XL* (*Mcl-1*^*Bcl-xL*^), or *A1* (*Mcl-1*^*A1*^), resulted in neurodevelopmental defects that could not be rescued by anti-apoptotic functions alone, providing evidence that the noncanonical functions of MCL-1 are indispensable for proper neurodevelopment^26^. Studies in the field have shown that disruption of the FAO pathway promotes asymmetric differentiation of adult mouse NSCs, highlighting FAO’s role in regulating stem cell fate decisions^36–39^. Although these studies have established a paradigm for the impact of MCL-1 on FAO and the impact of FAO on mouse adult NSCs, the effects of MCL-1 on the identity of human NSCs remain unknown. Further investigation of the specific non-apoptotic mechanisms of MCL-1 is essential for understanding its role in regulating the mitochondrial processes in human NSCs.

Work from the Opferman and Walensky groups revealed an unexpected link between MCL-1 and fatty acid oxidation (FAO) in cancer cells^22,24,25^. Inhibition of MCL-1 results in decreased oxidation of long-chain fatty acids (LCFAs) in cancer cells^22,24^, likely due to the disrupted interaction between MCL-1 and the BH3-like domain of acyl-CoA synthetase long chain family member 1 (ACSL-1). This suggests that MCL-1 is a direct modulator of FAO in cancer cells^25^; however, this has not been investigated in the context of human neurodevelopment.

FAO is a metabolic process critical for energy production and cellular homeostasis that occurs within the mitochondrial matrix and requires LCFAs to pass the OMM and IMM. Starting at the OMM, LCFAs are first activated to acyl-CoA derivatives by ACSL1 and then catalyzed by carnitine palmitoyltransferase I (CPT1) into a fatty acylcarnitine. At the IMM, carnitine palmitoyltransferase II (CPT2) functions to cleave the fatty acylcarnitine and release the fatty acyl-CoA for β-oxidation by Very Long Acyl-CoA Dehydrogenase (VLCAD) at the matrix. The enzymatic breakdown of LCFAs is a multistep process that produces acetyl-CoA, a metabolic byproduct which enters the tricarboxylic acid (TCA) cycle where it generates citrate that *exits* the mitochondria and can be used for histone acetylation^40,41^. Disruption of the FAO pathway promotes asymmetric differentiation of pluripotent and neural stem cells, suggesting a role of FAO in stem cell fate decisions^36–39,42^. Although FAO modulates neurodevelopment and MCL-1 is known to play a role in cancer cell FAO, the role of MCL-1 in FAO during human neurodevelopment has not been explored. Here, we report the non-apoptotic effects of MCL-1 inhibition on mitochondrial and cristae morphology and FAO in NSCs. Our findings provide evidence that MCL-1 maintains the integrity of mitochondrial cristae and neural progenitor cell identity.

## Results

### MCL-1 inhibition disrupts mitochondrial architecture and inner membrane organization in human neural stem cells

**Figure 1.**
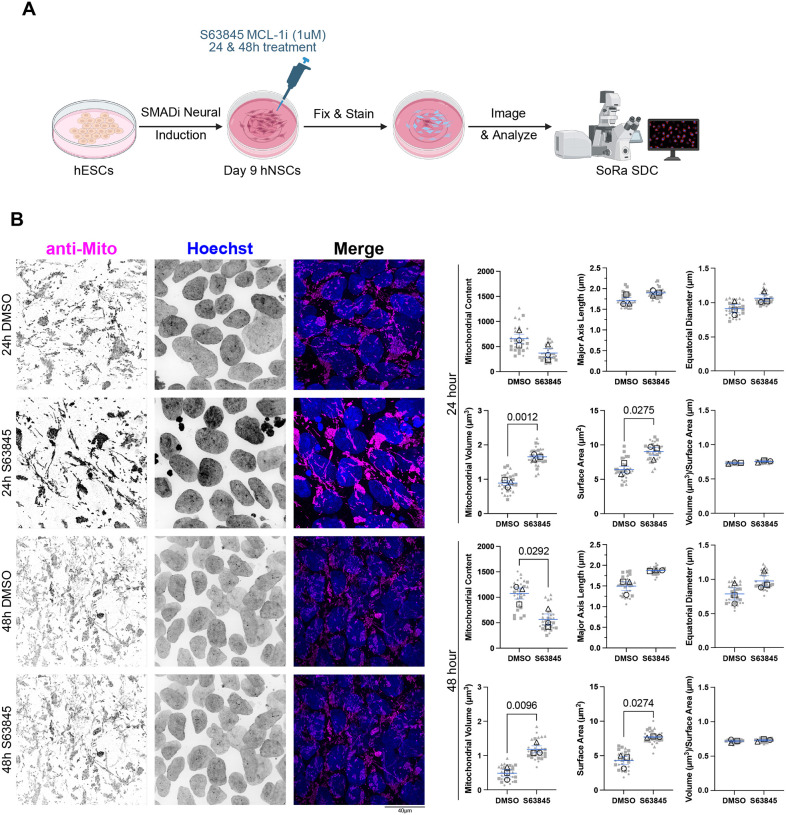
S63845 impacts mitochondrial content and morphology. (A) Schematic detailing the neural induction of human embryonic stem cells (hESCs) using SMAD inhibition (SMADi) for the derivation of the human neural stem cells (hNSCs) tissue culture model. hNSCs are maintained in neural induction media (NIM) with daily media changes until they are passaged on day 7. hNSCs are given a 24-hour acclimation period on day 8 before treatment on day 9 with 1μm S63845 (MCL-1 inhibitor), and DMSO as the control group. Samples were then collected at 24- and 48-hours post-treatment on days 10 and 11 after which cells were fixed and stained for imaging on a SoRa spinning disk confocal (SDC) microscope. (B) Immunofluorescent images of mitochondria (anti-mito in magenta) and nuclei (Hoechst in cyan) acquired on SoRA SDC at 100X (scale bar = 40μm). Representative images (n=3) of 24- and 48-hours post-treatment with DMSO control and S63845 (MCL-1 inhibitor). Quantification of mitochondrial content and average measures of mitochondrial morphology. Nine images were acquired per condition and plotted in gray. Each large, open-shape data point represents the mean per biological replicate. Shape of data point corresponds to each biological replicate (open circle for n=1, open square for n=2, and open triangle for n=3). Means of the biological replicates were analyzed using a parametric two-tailed unpaired t-test with a Welch’s Correction. Error bars represent standard error of the mean.

MCL-1 is known for its canonical function as an anti-apoptotic member of the BCL-2 family and is indispensable for the survival of human induced pluripotent stem cells^17^, hematopoietic stem cells^27,43^, and cardiomyocytes^23^. Similarly, embryonic neural precursor cells depend on MCL-1, as conditional knockout murine models, these cells display widespread apoptosis upon MCL-1 loss^44^. These findings suggest a general dependency on MCL-1 for the survival of neural precursor cells across developmental stages. To examine the effect of MCL-1 inhibition on hNSCs, we differentiated hESCs with SMAD inhibitors, as previously described^45^, and treated them with S63845, a selective BH3 mimetic inhibitor of MCL-1, for 24 or 48 hours (Figure 1A). Previous studies reported an increase in MCL-1 protein levels upon MCL-1 inhibition, which is speculated to be due to the stabilization of MCL-1 and protection from degradation by proteases when its BH3 domain is occupied^46^. Using RT-qPCR, we observed a downregulation of *MCL-1* at 24 hours of inhibition but not 48 hours (Supplementary Figure 1A). Co-treatment with Q-VD-OPh (QVD), a potent pan-caspase inhibitor, did not result in downregulation of *MCL-1* expression (Supplementary Figure 1B). As previously reported, we also observed an increase in MCL-1 protein levels in hNSCs at both timepoints (Supplementary Figures 1C–D) despite the downregulation of *MCL-1* expression at 24 hours. This increase in MCL-1 protein levels serves as an internal control that the S63845 small molecule inhibitor is effectively inhibiting MCL-1 in hNSCs. However, this upregulation was not observed when hNSCs were co-treated with QVD (Supplementary Figures 1E–F).

The mitochondrial network of hNSCs treated with S63845 at both timepoints exhibited a swollen mitochondrial network with greater volume and surface area as revealed by super-resolution microscopy (Figure 1B). MCL-1 inhibition at 48 hours resulted in a significant reduction in mitochondrial content, indicating progressive elongation and compaction of the mitochondrial network. The maximum mitochondrial volume and equatorial diameter revealed an expanded mitochondrial network at both timepoints (Supplementary Figure 2A). hNSCs co-treated with QVD do not exhibit these mitochondrial network changes (Supplementary Figure 2B).

**Figure 2.**
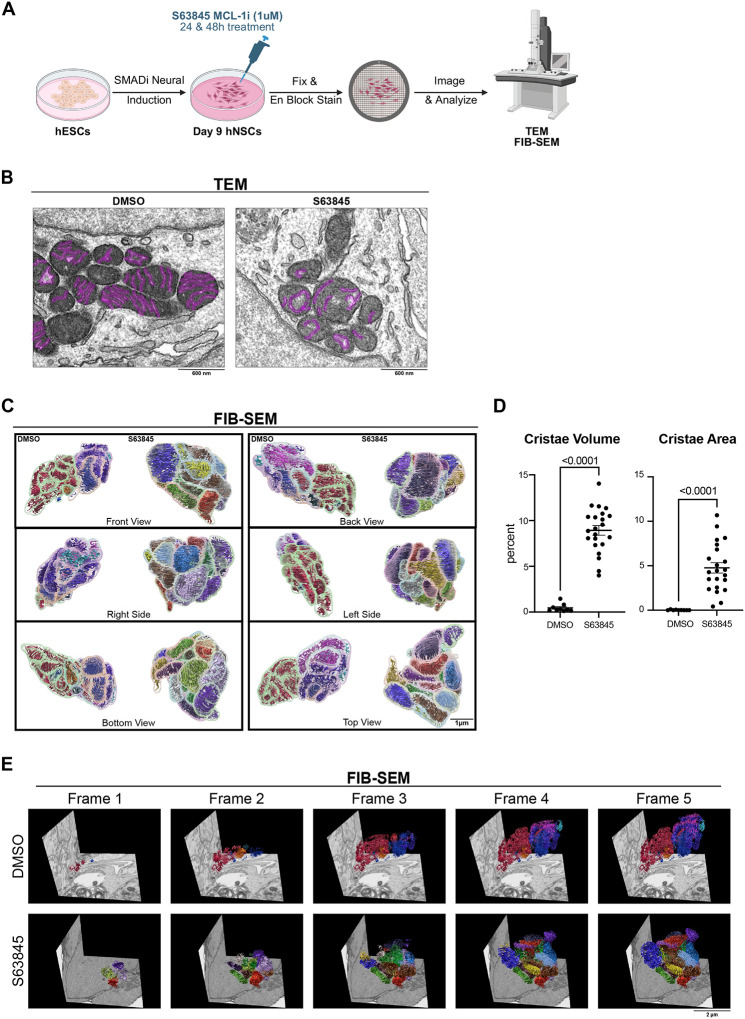
MCL-1 inhibition compromises mitochondrial cristae integrity. (A) Schematic detailing the neural induction of human embryonic stem cells (hESCs) using SMAD inhibition (SMADi) for the derivation of the human neural stem cells (hNSCs) tissue culture model. hNSCs are maintained in neural induction media (NIM) with daily media changes until they are passaged on day 7. hNSCs are given a 24-hour acclimation period on day 8 before treatment on day 9 with 1μm MCL-1 inhibitor (S63845 MCL-1i) with DMSO as the control group. Samples were then collected at 48-hours post-treatment on day 11 after which cells were fixed and processed for transmission electron microscopy (TEM) or focused ion beam-scanning electron microscopy (FIB-SEM). (B) Representative TEM images (n=3) of hNSC mitochondrial cristae 48-hours post-treatment with DMSO control and S63845 MCL-1 inhibitor. Scale bar = 600nm. (C) 3-dimensional reconstructions of mitochondria and cristae after 48-hour treatment with S63845 (MCL1 inhibitor) (scale bar = 0.5μm). (D) Quantification of cristae changes from 3-dimensional reconstruction. Each cristae volume and surface area was normalized to (divided by) the corresponding mitochondrion volume and surface area, respectively, and multiplied by 100 to get the percentage of change. Each data point shown is an individual mitochondrion. Data were analyzed using a parametric two-tailed unpaired t-test with a Welch’s correction. (E) Volumetric still frames of mitochondrial cristae 3-D reconstruction of NSCs at 48 hours post-treatment with DMSO control and S63845 (MCL-1 inhibitor) segmented from FIB-SEM volume images. Scale bar = 3000 nm.

Due to the critical role of mitochondrial cristae in metabolism regulation, we interrogated whether the effects of MCL-1 inhibition on mitochondrial morphology extended to the ultrastructure of the cristae using transmission electron microscopy (TEM) (Figure 2A). The hNSCs treated with MCL-1 inhibitor exhibited an atypical cristae morphology at 48 hours (Figure 2B). Three-dimensional FIB-SEM reconstruction further revealed an increase in cristae volume and surface area (Figures 2C–D) but not mitochondrial branching index (MBI) or sphericity (Supplementary Figure 3A). MCL-1 inhibition led to extensive loss of cristae organization in S63845-treated cells (Figure 2A–E). Compared to the densely folded membranes of control mitochondria, those from MCL-1–inhibited cells exhibited swollen matrices and sparse, disordered cristae. Volumetric analyses revealed a sharp increase in both cristae surface area and volume, underscoring the significance of MCL-1 for maintaining inner membrane complexity and structural integrity.

### MCL-1 activity preserves OPA1 proteostasis and MICOS complex stability

**Figure 3.**
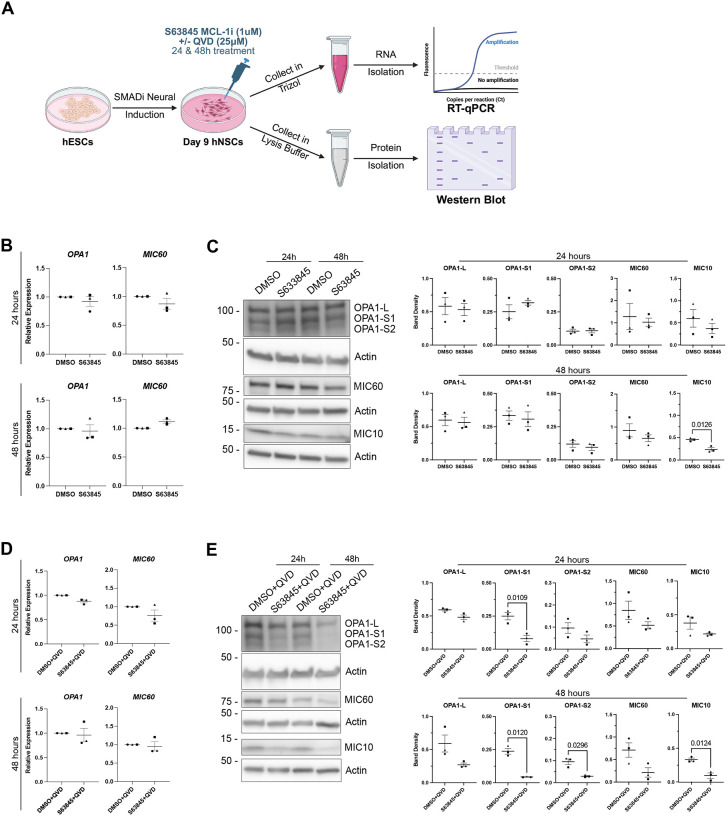
S63845 and QVD co-treatment reveal altered levels of cristae-shaping proteins. (A) Schematic detailing the neural induction of human embryonic stem cells (hESCs) using SMADi. hNSCs are maintained in neural induction media (NIM) with daily media changes until they are passaged on day 7. hNSCs are given a 24-hour acclimation period on day 8 before treatment on day 9 with 1μm S63845 (MCL-1 inhibitor), plus or minus 25 μM QVD co-treatment, with DMSO or DMSO+QVD as the control groups. Samples were then collected at 24- and 48-hours post-treatment on days 10 and 11, respectively. Cells were collected in either Trizol for real-time quantitative polymerase chain reaction (RT-qPCR) or protease inhibitor-containing lysis buffer for western blot. (B) Quantified relative expression of *OPA1* and *MIC60* after 24- and 48-hour treatments with DMSO control and S63845 (MCL-1 inhibitor). (C) Left panel: representative images (n=3) of OPA1-L, OPA1-S1, OPA1-S2, MIC60, and MIC10 after 24- and 48-hours of treatment with DMSO control and S63845 (MCL-1 inhibitor). Right panel: quantified band density. (D) Quantified relative expression of *OPA1* and *MIC60* after 24- and 48-hour S63845 plus QVD co-treatment. (E) Representative images (n=3) and quantified band density of OPA1-L, OPA1-S1, OPA1-S2, MIC60, and MIC10 after 24- and 48- hour S63845 plus QVD co-treatment. Each data point represents a biological replicate. Shape of data point corresponds to each biological replicate (circle for n=1, square for n=2, and triangle for n=3). Data analyzed using a parametric two-tailed unpaired t-test with a Welch’s Correction. Error bars represent standard error of the mean.

Given the pronounced inner membrane defects, we examined the proteins responsible for cristae shaping and junction formation (Figure 3A). The cristae structure is maintained by the mitochondrial contact site and cristae-organizing system (MICOS) complex, composed of the MIC60 subcomplex (MIC60, MIC19, MIC25) and the MIC10 subcomplex (MIC10, MIC26, MIC27) bridged together by MIC13/QIL^47^. The MICOS complex is responsible for establishing junctions that are required for cristae biogenesis in the IMM to increase its surface area for synthesis of adenosine triphosphate (ATP) via oxidative phosphorylation (OXPHOS) through the electron transport chain (ETC)^16,48^. The large GTPase, OPA1, contributes to the stabilization of the MICOS and cristae architecture^49^. The long form of OPA1 (OPA1-L) has a well-established role in IMM fusion^50^ and is also critical for the maintenance of cristae lumen width^51^. Cooperatively, the short form of OPA1 (OPA1-S) maintains the length of the cristae lumen^51^. We first examined whether MCL-1 inhibition alone or in combination with QVD alters the expression of *MIC60* or *OPA1* mRNA and found no significant fold change (Figure 3B–D). We detected a decrease of OPA1-S1 protein levels at 24 and 48 hours and of OPA1-S2 at 48 hours of MCL-1 plus caspase inhibition, but not with MCL-1 inhibition alone (Figures 3C–E). Thus, MCL-1 inhibition disrupts the balance between long (fusion-competent) and short OPA1 isoforms, hindering proteolytic cleavage of OPA1-L into smaller fragments. Immunoblotting analysis for MIC60 and MIC10, the core components of the MICOS subcomplexes, revealed a similar reduction in abundance of MIC10 protein levels at 48 hours of MCL-1 inhibition (Figure 3C). Co-treatment with the caspase inhibitor QVD did not rescue the levels of MIC10 (Figure 3E), indicating that OPA1 processing and MICOS destabilization are not a consequence of caspase activation. We did not observe a change in other MICOS protein levels (Figure 3C and Supplementary Figure 3B). These data identify MCL-1 as a critical regulator of mitochondrial inner membrane organization, acting upstream of OPA1- and MICOS-dependent cristae maintenance.

### MCL-1 inhibition impairs FAO and metabolic coupling.

**Figure 4.**
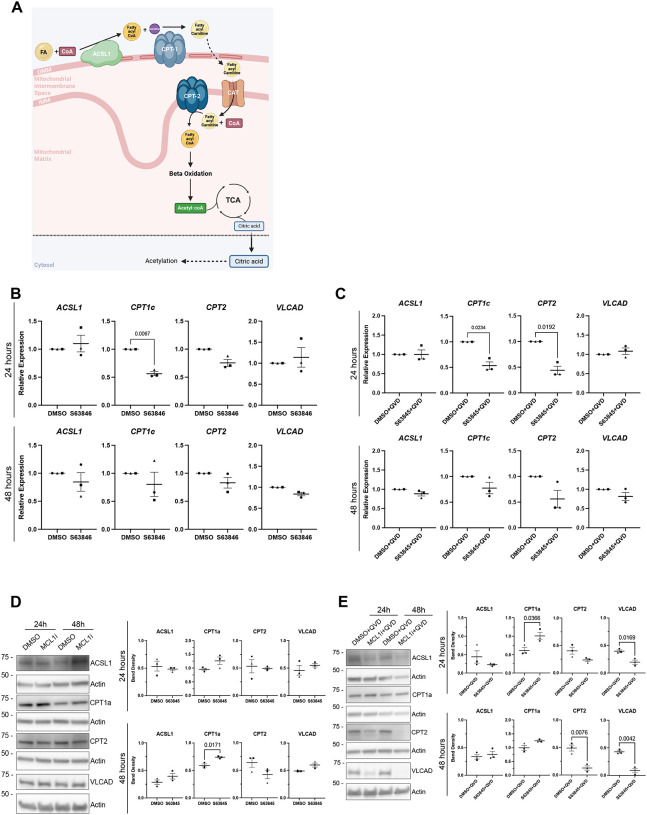
S63845 and QVD co-treatment results in downregulation of carnitine palmitoyltransferase and altered levels of FAO enzymes. (A) Schematic of the mitochondrial fatty acid beta-oxidation pathway (B) Quantified relative expression of FAO enzymes after 24- and 48-hours post-treatment with DMSO and S63845 (MCL-1 inhibitor). (C) Quantified relative expression of FAO enzymes after 24- and 48-hour S63845 plus QVD co-treatment. (D) Representative images (n=3) and quantified band density of ACSL1, CPT1a, CPT2, and VLCAD after 24- and 48-hours of treatment with DMSO control and S63845 (MCL-1 inhibitor). (E) Representative images (n=3) and quantified band density of ACSL1, CPT1a, CPT2, and VLCAD after 24- and 48- hour S63845 plus QVD co-treatment. Each data point on the graph represents a biological replicate. Shape of data point corresponds to each biological replicate (circle for n=1, square for n=2, and triangle for n=3). Data analyzed using a parametric two-tailed unpaired t-test with Welch’s Correction. Error bars represent standard error of the mean.

We predict that the transmembrane FAO enzymes and transport proteins heavily rely on the proper assembly and structure of the OMM and IMM. Previous studies have shown a close interaction between MCL-1 and FAO enzymes in cancer cells^22,25^. A previous study reported a direct interaction with MCL-1 and the BH3-like domain of ACSL1, which is the enzyme responsible for activating fatty acids to fatty acyl CoA in the first step of the FAO pathway (Figure 4A). Mouse studies also established the importance of FAO for maintenance of adult NSCs^37,42^. To explore whether the mitochondrial structural perturbations extend to mitochondrial function, we assessed key regulators of fatty acid β-oxidation. Expression analyses revealed no changes in FAO enzyme transcripts with MCL-1 inhibition alone (Figure 4B), but a significant downregulation of *CPT2* transcripts at 24 hours following MCL-1 and caspase inhibition, while *ACSL1* and *CPT1a* levels remained stable (Figure 4C). Increased levels of CPT1a were detected at 48 hours following inhibition of MCL-1 (Figure 4D). Furthermore, co-treatment with caspase inhibition revealed an increase of CPT1a levels earlier at 24 hours, along with a decrease of CTP2 at 48 hours and VLCAD at both timepoints (Figure 4E). The alteration in β-oxidation components persisted despite caspase inhibition, confirming a direct metabolic effect of MCL-1 loss. These findings suggest that MCL-1 supports mitochondrial lipid utilization by sustaining the expression of enzymes critical for fatty acid transport and oxidation.

**Figure 5.**
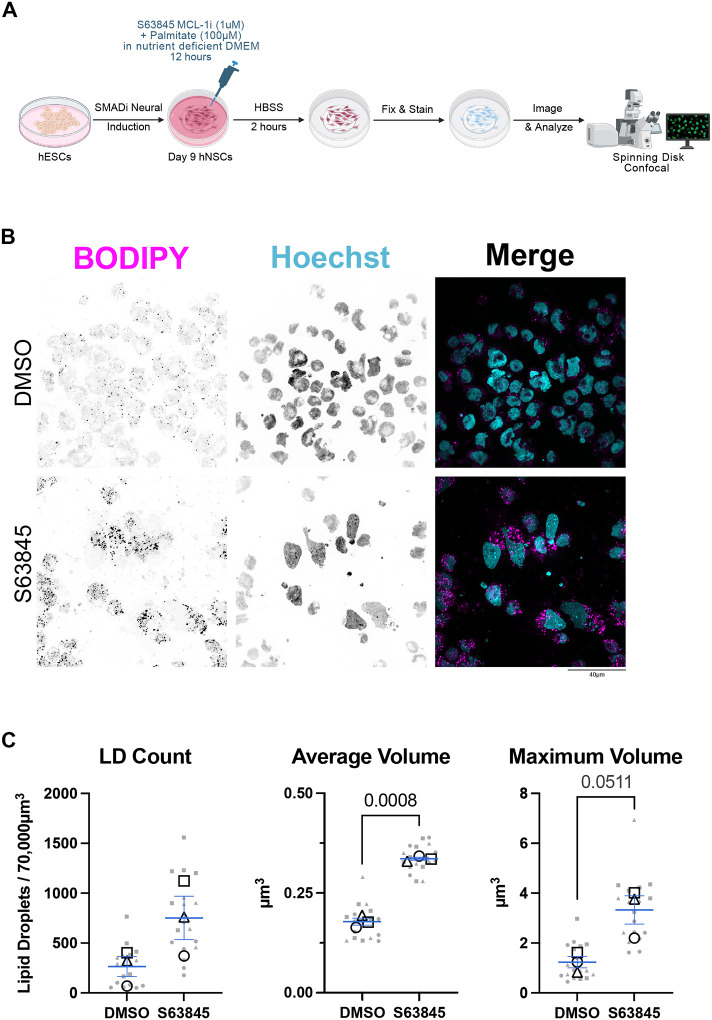
hNSCs supplemented with palmitate and treated with S63845 accumulate lipid droplets. (A) Schematic of experimental design. On day 9 post-induction, hNSCs were treated with S63845 in nutrient deficient DMEM media (-glucose, -glutamine, -pyruvate) and supplemented with 100μM of palmitate for 12 hours followed by a 2-hour starvation in HBSS. Cells were then live stained with BODIPY and Hoechst prior to PFA fixation. (B) Representative fluorescent images (n=3) of lipid droplets (BODIPY in magenta) and nuclei (Hoechst in cyan) were acquired on a spinning disk confocal microscope at 100X (scale bar = 40μm). (C) Quantification of lipid droplet content, average volume, and maximum volume. Six images were acquired per condition and plotted in gray with shape corresponding to each biological replicate. Each open-shape data point represents the mean per biological replicate. Shape of data point corresponds to each biological replicate (open circle for n=1, open square for n=2, and open triangle for n=3). Means of the biological replicates were analyzed using a parametric two-tailed unpaired t-test with a Welch’s Correction. Error bars represent standard error of the mean.

Fatty acids get stored in lipid droplet organelles when they are not utilized for β-oxidation. Accumulation of lipid droplets upon MCL-1 inhibition was previously reported in cancer cells as a marker of disruption in β-oxidation^25^. To further investigate the impact of MCL-1 on FAO, hNSCs were cultured in nutrient-deficient DMEM media supplemented only with palmitate during treatment with MCL-1 inhibitor, followed by a 2-hour starvation period in HBSS and S63845 (Figure 5A). Consistent with impaired β-oxidation, S63845-treated hNSCs accumulated excess neutral lipids when challenged with palmitate as their only resource for fuel (Figure 5B). Quantitative imaging revealed increased lipid droplet average and maximum volume (Figure 5C), indicative of reduced lipid turnover and a shift toward storage.

### MCL-1 maintains neural progenitor identity and supports neuronal differentiation

**Figure 6.**
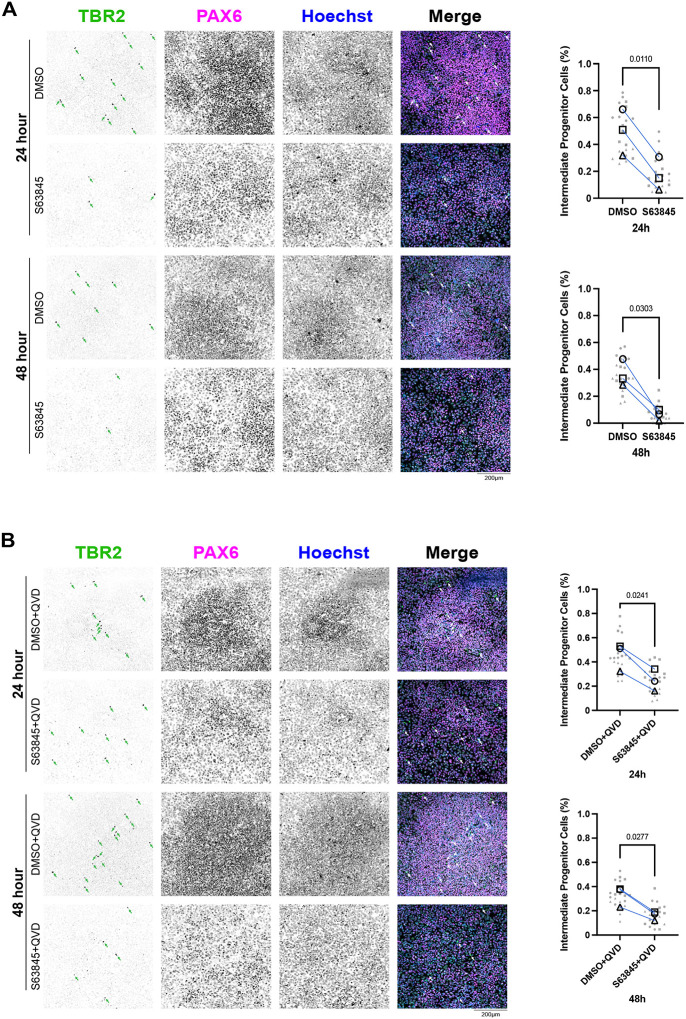
S63845 results in depletion of TBR2^+^/PAX6^−^ IPCs independent of cell death. Representative fluorescent images (n=3) of nuclear identity markers TBR2 (green), PAX6 (magenta), and nuclei (Hoechst in blue) were acquired on a spinning disk confocal microscope at 20X (scale bar = 200μm). Intermediate progenitor cells (IPCs) identified by presence of TBR2 and absence of PAX6. (A) Quantified percentage of IPCs after 24- and 48-hour treatments with S63845 (MCL-1 inhibitor). (B) Quantified percentage of IPCs after 24- and 48-hour S63845 plus QVD co-treatment. Quantification of IPCs manually counted and normalized to total cell count for final percentage output. Seven images were acquired per condition and plotted in gray. Each large, open-shape data point represents the mean per biological replicate. Shape of data point corresponds to biological replicate (open circle for n=1, open square for n=2, and open triangle for n=3). Mean of biological replicates analyzed using a two-tailed paired parametric t-test.

**Figure 7.**
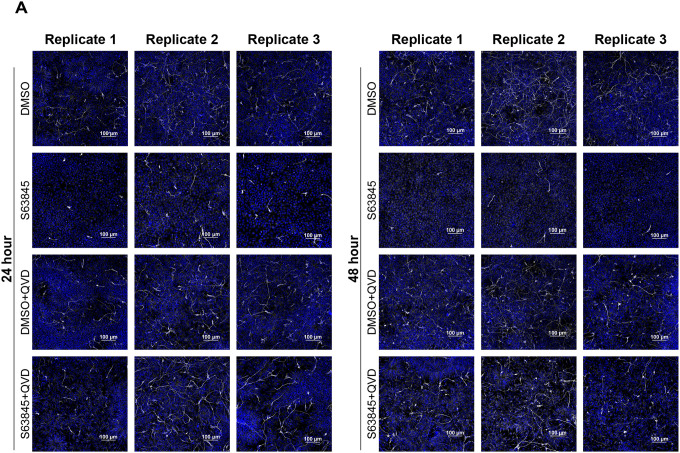
S63845 results in depletion of BIII-Tubulin^+^ newborn neurons. (A) Representative fluorescent images of each biological replicate (n=3) of hNSCs treated with S63845 alone or with QVD co-treatment. Newborn neurons labeled with BIII-Tubulin (white) and nuclei (Hoechst in blue) were imaged on a spinning disk confocal microscope at 60X (scale bar = 100μm).

Given the role of MCL-1 in FAO and the mitochondrial and metabolic perturbations triggered by MCL-1 inhibition, we investigated the effects of MCL-1i treatment on neurogenic progression. Although we did not detect changes in protein levels of hNSC identity markers (Supplementary Figure 4A), we observed a significant downregulation of *PAX6* (a marker of neural stem cell identity) and *EOMES* (the gene encoding TBR2, a marker of intermediate progenitor cells (IPCs)) at 24 hours that was not rescued by QVD co-treatment and persisted into 48 hours with co-treatment (Supplementary Figure 4B). Immunofluorescent imaging revealed a small population of IPCs (PAX6^−^/TBR2^+^) in the culture of hESCs induced with dual SMAD inhibition. IPCs are committed progenitor cells that give rise to glutamatergic projection neurons. MCL-1 inhibition selectively reduced the proportion of PAX6^−^/TBR2^+^ IPCs while sparing PAX6^+^ radial glia–like progenitors (Figure 6A). This loss of IPCs persisted under caspase inhibition (Figure 6B), suggesting that MCL-1 is critical for maintaining the identity of this progenitor pool through non-apoptotic mechanisms. To determine how these effects on IPCs impact neuronal differentiation, we first examined the ability of the cells to proliferate using EdU labeling and found no significant changes in late or early S-phase (Supplementary Figure 5A&B). To further investigate the potency of neural stem/progenitor cells we examined their ability to differentiate and give rise to newborn neurons using βIII-tubulin staining (Figure 7). Control cells formed dense neurite networks with extensive βIII-tubulin labeling, whereas MCL-1–inhibited neurons displayed sparse and truncated processes, reflecting defective neurite extension and maturation (Figure 7). When treated with MCL-1 inhibitor, neural stem/progenitor cells gave rise to less newborn neurons with compromised projections that lacked complexity but that was rescued upon co-treatment with QVD. This sheds light on the importance of MCL-1 for the potency of neural stem/progenitor cells to undergo proper differentiation and for newborn neurons to undergo proper maturation (Figure 7B). The absence of overt cytotoxicity suggests that MCL-1 directly supports neuronal commitment rather than survival alone.

## Discussion

Although MCL-1 has been traditionally studied as an anti-apoptotic BCL-2 family member^21,52^, accumulating evidence supports its critical role in maintaining mitochondrial homeostasis across diverse cell types^25,26^. Our findings extend this paradigm to the developing human brain. By combining ultrastructural, molecular, and functional analyses, we report that MCL-1 sustains the integrity of mitochondrial architecture and its functional coupling to metabolic flux. Inhibition of MCL-1 leads to structural collapse of the cristae and disassembly of the MICOS complex as well as OPA1, two scaffolds required for cristae maintenance. Consequently, MCL-1 inhibition impairs FAO metabolism, as evidenced by altered FAO enzyme levels and lipid droplet accumulation. We propose that efficient FAO depends on both cristae surface area and the localization of β-oxidation enzymes to defined subdomains of the inner membrane. These perturbations are accompanied by defects in IPC maintenance and neuronal maturation. Our findings establish a non-apoptotic role for MCL-1 in sustaining mitochondrial organization and metabolic competence, which are indispensable for proper human neurogenesis. Taken together, the data suggest that MCL-1 integrates mitochondrial architecture with metabolic capacity to support progenitor identity.

While MCL-1 has been reported to influence FAO in cancer cells and to localize to the IMM, its impact on cristae integrity has remained unexplored until now. Our findings bridge this gap by revealing that MCL-1 is indispensable for maintaining cristae organization and metabolic FAO competence in hNSCs. The observed reduction in mitochondrial content upon MCL-1 inhibition likely reflects a compensatory failure where mitochondria are increased in volume and surface but lose metabolic function as apparent by lipid droplet accumulation. We find this disruption to be coupled to a decline in FAO enzymes. Further investigation is required to determine whether ACSL1 localization is affected by MCL inhibition in hNSCs, as reported in cancer cells. Whether ACSL1 integrates into the OMM or needs only to be in close proximity to the OMM for the activation of LCFAs is not known. Given that the altered FAO enzyme levels were all downstream of ACSL1, this raises the question of whether ACSL1 is a rate-limiting step in the mitochondrial beta-oxidation pathway. If ACSL1 is displaced from the mitochondria, this may disrupt a feedback loop that maintains the expression and stabilization of FAO enzymes, thereby dismantling the FAO signaling pathway and leading to lipid oxidation dysfunction. The alterations in FAO enzyme expression and protein levels accompanied by mitochondrial swelling suggest that MCL-1 coordinates spatial coupling between lipid activation and oxidative metabolism.

Given that efficient FAO is a defining feature of the progenitor metabolic state, this association could represent a key mechanism linking mitochondrial structure and function to the maintenance of stem cell fate. The influence of mitochondrial function on developmental fate decisions is increasingly recognized. *In vivo* and *in vitro* studies have identified a postmitotic window of neurogenic plasticity directed by mitochondrial morphology^44,35,53^. Moreover, disruption of the FAO pathway promotes asymmetric differentiation of adult mouse pluripotent and neural stem cells, signifying a role of FAO in stem cell fate decisions^37,42^. Our data reveal that MCL-1 inhibition disrupts this balance. MCL-1 inhibition does not alter hNSC proliferation, but instead results in reduced intermediate progenitors and impaired neuronal differentiation and maturation. Bioenergetically demanding cells must meet their metabolic needs for proper function and survival. The metabolic intermediates of mitochondrial FAO, such as acetyl-CoA, may alter gene expression via histone modifications (e.g., acetylation) and ultimately influence cell fate decisions. In the delicate process of development, high-energy-demanding cells must maintain a specific metabolic profile for survival, making metabolism a quality-control checkpoint. If a cell does not meet the criteria for differentiation, its fate shifts to maintain tissue integrity.

A key question emerging from this study is whether MCL-1 directly regulates FAO through physical association with lipid import enzymes or indirectly by maintaining cristae topology. We hypothesize that MCL-1 associates with both MICOS and FAO enzymes at discrete cristae contact points, thereby coupling membrane morphology to metabolic signaling. These results position MCL-1 upstream of the structural regulators OPA1 and MICOS. In alignment with our observations, mutations in cristae-shaping proteins are associated with severe neurodevelopmental disorders, reinforcing the notion that mitochondrial ultrastructure is not only permissive but also instructive in fate specification.

Taken together, our results define a previously unappreciated dimension of MCL-1 function in human neurogenesis, albeit using an *in vitro* experimental paradigm. Beyond its canonical anti-apoptotic role, MCL-1 emerges as a structural and metabolic hub that links cristae architecture, lipid oxidation, and lineage progression. We propose a model in which MCL-1 associates with MICOS and FAO complexes to maintain cristae organization and metabolic flux, thereby sustaining the progenitor state and enabling efficient neuronal differentiation. Future studies dissecting the molecular interfaces between MCL-1, MICOS proteins, and FAO enzymes will be critical for determining whether MCL-1 acts as a scaffold that integrates mitochondrial structure and metabolic signaling. Understanding this connection could illuminate fundamental mechanisms by which mitochondrial morphology encodes developmental potential and may reveal new vulnerabilities in neurodevelopmental and degenerative disorders.

## Methods

### Human Embryonic Stem Cells (hESCs)

Human embryonic stem cell line, H9 (WA09), was obtained from WiCell Research Institute (Wisconsin). H9s were maintained in mTeSR (STEMCELL Technologies, cat # 85850) media on 6-well plates coated with Matrigel (Corning, cat # 354277) at 37°C with 5% CO2. Culture medium was changed daily. Cells were checked daily for differentiation and were passaged every 3–4 days using Gentle cell dissociation solution (STEMCELL Technologies, cat # 07174). All experiments were performed under the supervision of the Vanderbilt Institutional Human Pluripotent Cell Research Oversight (VIHPCRO) Committee. Cells were periodically checked for contamination.

### hESC-derived hNSC culture

H9s were cultured until reaching 70–80% confluency, ensuring minimal differentiation at the colony edges. Cells were dissociated using 1 mL of a Gentle Cell Dissociation Reagent (STEMCELL Technologies, cat # 100–0485) and incubated at 37°C for 8 minutes. 2 mL of DMEM-F12 (Thermo Scientific, cat # 11320033) supplemented with 10 μM of ROCK inhibitor (STEMCELL Technologies, cat # 72307) was added to each well. Cells were gently scraped, pipetted for transfer into a conical tube, and centrifuged at 300 × g for 5 minutes. Cell pellet was resuspended in STEMdiff^™^ SMADi Neural Induction Medium (NIM) (STEMCELL Technologies, cat # 08581) supplemented with 10 uM ROCK inhibitor. Cell suspension was counted and plated at a density of 2.0–2.5×10^6^ cells per well in a 6-well plate. Cells were incubated at 37°C for 7 days, with daily feeding of NIM.

### Western blot

Cells were harvested in lysis buffer containing 1% Triton X-100 (Sigma, cat # T9284), 1X complete protease inhibitor cocktail (Roche, cat # 04693159001), 1X PhosSTOP phosphatase inhibitor (Roche, cat # 04906837001), and 0.5mM PMSF (RPI, cat # P20270). Samples were kept on ice and vortexed for 30 seconds every 10 minutes. Lysates were centrifuged at 14,100 × g for 30 minutes at 4°C. Supernatant was collected and protein concentrations were quantified using the Pierce BCA Protein Assay Kit (ThermoScientific, cat # 23225). Western blotting was performed using the Bio-Rad Mini-PROTEAN Electrophoresis System (Bio-Rad, cat # 1658036). Samples were run on 4–20% Mini-PROTEAN TGX gels (Bio-Rad, cat # 4561095). Protein was transferred from gels onto methanol activated PVDF membranes (Bio-Rad, cat # 1620177). All membranes were washed in Tris-buffered saline with 0.1% Tween-20 (TBST) and blocked in TBST containing 5% milk for 1 hour at room temperature. Primary antibodies were incubated at listed dilution in TBST containing 5% milk overnight at 4°C. Secondary antibodies were used at listed dilution in TBST containing 5% milk and incubated for 1 hour at room temperature. Protein signal was developed using either Pierce ECL Western Blot Substrate (32109, ThermoScientific) or SuperSignal West Femto (34094, ThermoScientific), depending on the sensitivity of the primary antibody. Protein signal was visualized using the Amersham Imager 600 (29-0834-61, GE Life Sciences). Acquired images were quantified using Thermo Fisher Scientific iBright Analysis Software and representative images were composed using Affinity Designer 2.

**Table:** 

Antibody	Source	Catalogue Number	RRID	Dilution	Application
Mouse antimitochondria	Abcam	ab92824	AB_10562769	1:200	Immunofluorescence
Mouse anti-PAX6	BD Pharmingen	561462	AB_10715442	1:200	Immunofluorescence
Rabbit anti-EMOES (TBR2)	Cell Signaling Technology	81493	AB_2799974	1:200	Immunofluorescence
Mouse anti-b-Actin	Sigma-Aldrich	A1978	N/A	1:10,000	Western blot
Rabbit anti-cleaved PARP	Cell Signaling Technology	5625S	AB_10699459	1:1000	Western blot
Rabbit anti-MCL-1	Cell Signaling Technology	94296S	AB_2722740	1:1000	Western blot
Rabbit anti-CHCHD3 (MIC19)	Proteintech	25625-1-AP	AB_2687533	1:10,000	Western blot
Rabbit anti-IMMT (MIC60)	Sigma-Aldrich	HPA036164	AB_2674979	1:5000	Western blot
Rabbit anti-OPA-1	Cell Signaling Technology	67589S	AB_2799728	1:5000	Western blot
Rabbit anti-PAX6	Cell Signaling Technology	60433S	AB_2797599	1:500	Western blot
Mouse anti-SOX2	Abcam	ab79351	AB_10710406	1:500	Western blot

### RNA extraction and cDNA synthesis

Cells were collected in 1000 μl TRIzol reagent. 200 μl of chloroform was added and the samples were incubated at room temperature for 5 minutes prior to centrifugation at 12,000 × g at 4°C. The aqueous phase was collected and 500 μl of isopropanol was added to precipitate RNA. Samples were incubated for 25 minutes at room temperature, followed by centrifugation at 12,000 × g for 10 minutes at 4°C. The RNA pellet was washed with 75% ethanol, semi-dried, and resuspended in 30 μl of DEPC-treated water. Concentration was measured and 2 μg per sample was treated with DNAse (New England Biolabs #M0303) prior to generating cDNA using the manufacturer’s protocol (Thermo Fisher #4368814).

### Quantitative PCR (RT-qPCR)

50 ng of cDNA sample was used to run RT-qPCR using 20 μM of the primers listed in the table. QuantStudio 3 Real-Time PCR machine, SYBR green master mix (Thermo Fisher #4364346), and manufacturer instructions were used to set up the assay.

### Immunofluorescence

Cells were plated on 35 mm glass-bottom dishes (glass thickness of 0.16–0.19 mm) for confocal spinning disk immunofluorescence. Cells were fixed with 4% paraformaldehyde for 20 min and permeabilized in 1% Triton-X-100 for 10 min at room temperature. After blocking in 10% BSA for 1 hour, cells were treated with primary and secondary antibodies using standard methods. Primary antibodies were incubated at listed dilution in 10% BSA overnight at 4°C. Secondary antibodies were used at listed dilution in 10% BSA and incubated for 1 hour at room temperature. The nuclear stain used was HOECHST (Thermo Scientific, cat # H3570) and cells were mounted in Fluoromount-G (Electron Microscopy Sciences #17984–25) prior to imaging. Cells were labeled for EdU using the manufacturer’s protocol (Thermo Fisher #C10340). Lipid droplet staining was done live using BODIPY (Cayman Chemical Company #25892). Calls were incubated at 37°C for 15 minutes in 4 μM BODIPY prior to fixation in 4% PFA.

### Spinning Disk Confocal image acquisition

Images were acquired using a Nikon Spinning Disk Confocal outfitted with a Yokogawa CSU-X1 spinning disk head, Andor DU-897 EMCCD camera, and high-speed piezo [z] stage. All 100X images were acquired with a step size of 0.2 μm, while 20X images were acquired with a step size of 2 μm. Image processing and quantification was performed using NIS Elements (Nikon) and all representative images were generated using Fiji.

For mitochondrial morphology, we segmented mitochondria in 3D and performed skeletonization of the resulting 3D mask. Skeleton major axis length, volume, and surface area measurements were exported into GraphPad Prism v9.

### Tissue Preparation for TEM and FIB-SEM

Neural progenitor cells were plated at 1.5×10^6^ cells per 35 mm dish on Matrigel. The cells were washed with 1X PBS (3X), fixed in 2.5% glutaraldehyde in 0.1 M cacodylate prewarmed to 37°C, left to cool down at room temperature for 1hr, and placed at 4°C for an additional 24–48 hour of fixation. Cells were washed from fixative with 1X PBS (3x). Samples were sequentially post-fixed using the OTO method: 1.5% potassium ferrocyanide, 1% OsO4 in 0.1 M cacodylate for 30 minutes on ice, then 1% thiocarbohydrazide for 10 minutes, followed by 1% OsO4 in ddH2O for 10 minutes. Samples are then enblock stained with 2% uranyl acetate followed by lead aspartate. The samples were then dehydrated in a graded ethanol series and infiltrated with Durcupan resin using acetone as the transition solvent. The resin was polymerized at 60°C for 48 hours. For TEM, sections were cut on a Leica UC7 at a nominal thickness of 50 nm with no post-section staining. For FIB-SEM, samples were prepared as previously described^54^.

### TEM image acquisition and Cristae Scoring

Sections were imaged with either a ThermoFisher Scientific Tecnai T12 operating at 100 keV using an AMT nanosprint5 CMOS camera or a JEOL 2100+ operating at 200 keV using an AMT nanosprint15 II CMOS camera. In both cases, AMT acquisition software was used for imaging. The TEM images were analyzed using cristae scoring (Lam et. Al, 2021)^55^. The files were first renamed to blind the scoring process. The images were then blindly scored. A score was provided for each mitochondrion based on the quality of the cristae. The scoring rubric was as follows: 0 - there were no crista, 1 - more than 50% of the mitochondrial area did not have cristae, 2 - more than 25% of the mitochondria was without cristae, 3 - lots of cristae but irregular in structure, and 4 - many cristae that are regular structures.

### FIB-SEM image acquisition

Sections were imaged with Zeiss Crossbeam 550 FIB-SEM using ATLAS5 software for automated volumetric acquisition. Sample blocked were polished with a diamond knife and coated with platinum. Platinum pads were deposited via GIS on ROIs of interest, followed by milling in tracking marks and coating with GIS carbon. Milling was performed using a 700 pA Ga beam at 8 nm slice depth, imaging was performed 1.5 keV electron beam using the in column ESB detector for imaging at 8 nm pixels to make isotropic voxels. Volumetric slice alignments were performed in ATLAS5 using the two-window alignment method.

### FIB-SEM mitochondrial image analysis

FIB-SEM stacks were first cropped using Fiji is just Image J (FIJI). In the FIB-SEM stacks, none of the cells imaged were able to be visualized completely. Therefore, the crop size and location were selected by choosing a cell with bright, visible mitochondria. The crop was 500 × 500 pixels with a 3,000 nm depth. The cropped image was then imported into the FIJI LabKit plugin for segmentation^56^. For segmentation, each mitochondrion was manually traced with a separate label ID. Quality control was then performed following each mitochondrion through the entirety of the Z-stack and tracking all locations where the membranes connect. Any mitochondria that merged in any of the frames were labeled with the same label ID. The cropped FIB-SEM image and the proofread segmentation masks were imported into the IMARIS software. Ortho slices in the XY and XZ planes were added, and the proofread mask was selected to be identified by ID. This identifies each separate mitochondrion. Any mitochondria that were still split were manually combined in the IMARIS software using the unify feature. A clipping plane was then added. Animations were made using the animations tab in IMARIS with 50 frames per second and 1,000 frames total. Still images were captured using the screenshot feature. Volume quantification was done by volume measurement provided in the statistics tab in IMARIS. Surface area quantification was manually calculating using volume and sphericity measurements from the IMARIS statistics tab using the equation: $\frac{\pi^{\frac{1}{3}}{\left(6*\text{Volume}\right)}^{\frac{2}{3}}}{\text{sphericity}}$ Lastly, the mitochondrial branching index (MBI), was calculated by obtaining the BoundingBox00 length A (longest side) and BoundingBox00 length C (Shortest side) in the Vantage 2D tab and then dividing Length C/ Length A manually^57^.

### FIB-SEM cristae image analysis

The same FIB-SEM cropped images were used for the segmentation of the cristae. The cropped images were imported into FIJI and applied Contrast Limited Adaptive Histogram Equalization (CLAHE) to increase contrast. They were then imported into the LabKit plugin for segmentation. Each crista was manually traced, and all the cristae within one mitochondrion were labeled with the same label ID. Quality control was then performed, ensuring each mitochondrion had all its cristae labeled with the same ID. The cropped FIB-SEM image and the proofread segmentation masks were imported into the IMARIS software. Ortho slices in the XY and XZ planes were added, and the proofread mask was selected by ID. This identifies each separate mitochondrion’s cristae. Any errors in the cristae mask were merged using the unify feature in IMARIS. A clipping plane was then added to show the mitochondria throughout the entire z-plane. Animations were made using the animations tab in IMARIS with 50 frames per second and with 1,000 frames total. Still images were taken using the snapshot feature. Volume quantification was done by volume measurement provided in the statistics tab in IMARIS. Surface Area quantification was manually calculated using volume and sphericity measurements from IMARIS statistics tab using the equation: $\frac{\pi^{\frac{1}{3}}{\left(6*\text{Volume}\right)}^{\frac{2}{3}}}{\text{sphericity}}$. Cristae measurements were then normalized to the corresponding mitochondria by dividing the crista measurement by the mitochondrion measurement (i.e., $\frac{\text{Crista Volume}}{\text{Mitochonrion Volume}}$ The mitochondria segmentation was then added to the same IMARIS file as a second surface. The label was chosen by ID, and the surface type was changed to transparent to show the cristae within those mitochondria. Images were taken using the snapshot function, and animations were made with 50 frames per second and 1,000 frames total.

### Statistical analysis

All experiments were performed with at least 3 biological replicates. Statistical significance was determined by one-way, repeated measures one-way ANOVA, or t-test as appropriate for each experiment. Significance was assessed using Fisher’s protected least significance difference test. GraphPad Prism v9 was used for all statistical analysis and data visualization. Error bars in all bar graphs represent the standard error of the mean unless otherwise noted in the figure. No outliers were removed from the analyses. For all statistical analyses, a significant difference was accepted when P < 0.05.

## Supplementary Material

Supplement 1
