# Supplementary material for: Pharmacological inhibition of MCL-1 disrupts mitochondrial cristae and depletes the human neural progenitor cell pool
Source: bioRxiv. 2025 Dec 22:2025.12.12.694056. Originally published 2025 Dec 13. Preprint. [Version 2] doi: 10.64898/2025.12.12.694056 (PMC12713752; doi:10.64898/2025.12.12.694056)
Supplement: Supplement 1 [file media-1.pdf]

Supplementary Figure Legends

Supplementary Figure 1. S63845 effectively inhibits MCL-1 in hNSCs.

(**A**) Quantified relative expression of *MCL-1* after 24- and 48-hour treatment with S63845 MCL-1 inhibitor. (**B**) Quantified relative expression of *MCL-1* after 24- and 48-hour co-treatment with S63845 MCL-1 inhibitor and QVD. (**C**) Representative images (n=3) and (**D**) quantified band density of MCL-1 after 24- and 48-hours of treatment with S63845 MCL-1 inhibitor. (**E**) Representative images (n=3) and (**F**) quantified band density of MCL-1 after 24- and 48-hours of co-treatment with S63845 MCL-1 inhibitor and QVD. Each data point on graphs represents a biological replicate. Shape of data point corresponds to each biological replicate (circle for n=1, square for n=2, and triangle for n=3). Data analyzed using a parametric two-tailed unpaired t-test with Welch's Correction and a 95% confidence level (p-value ≤ 0.05 as the significance threshold). Error bars represent standard error of the mean.

Supplementary Figure 2. Maximum and minimum measurements of mitochondrial morphology.

(**A**) Quantification of mitochondria per cell and maximum measures of morphology – except for the minimum measurement of sphericity (volume/surface area). Mitochondria in magenta and nuclei (Hoechst) in blue. (**B**) Immunofluorescent images of mitochondria (anti-mito in magenta) and nuclei (Hoechst in cyan) acquired on SoRA SDC microscope at 100X (scale bar = 40μm). Representative images (n=3) of 24- and 48-hours after co-treatment with S63845 MCL-1 inhibitor and QVD. Quantification of mitochondrial content and average measures of mitochondrial morphology. Mitochondria per cell and maximum measures of morphology – except for the minimum measurement of sphericity (volume/surface area). Ten images were acquired per condition and plotted in gray. Each large, open-shape data point represents the mean per biological replicate. Shape of data point corresponds to biological replicate (open circle for n=1, open square for n=2, and open triangle for n=3). Means of the biological replicates were analyzed using a parametric two-tailed unpaired t-test with a Welch's Correction. Error bars represent standard error of the mean.

Supplementary Figure 3. Inhibition of MCL-1 disrupts mitochondrial cristae architecture

(**A**) Left panel: FIB-SEM 3-dimensional reconstruction of mitochondria after hNSCs are treated with S63845 MCL inhibitor for 48h (scale bar = 3000 nm). Right panel: quantified mitochondrial branching index (MBI) and Sphericity. (**B**) Representative images (n=3) and quantified band density of MICOS proteins MIC25, MIC13, and MIC19 after 24- and 48-hours of treatment with S63845 MCL-1 inhibitor alone (top panel) and QVD co-treatment (bottom panel). Each data point represents a biological replicate. Shape of data point corresponds to biological replicate (circle for n=1, square for n=2, and triangle for n=3). Data analyzed using a parametric two-tailed unpaired t-test with Welch's Correction and a 95% confidence level (p-value ≤ 0.05 as the significance threshold). Error bars represent standard error of the mean.

Supplementary Figure 4. Downregulation of key NSC identity markers caused by MCL-1 inhibition persists in the absence of caspase-mediated cell death.

(**A**) Representative images (n=3) and quantified band density of hNSC identity markers, PAX6 and SOX2 after treatment with S63845 MCL-1 inhibitor alone and QVD co-treatment for 24-hours (top panel) and 48-hours (bottom panel). (**B**) Quantified relative expression measured by RT-qPCR of identity markers, *PAX6*, *SOX2*, and *EOMES*, after 24- (top left) and 48-hour (bottom left) treatments with S63845 MCL-1 inhibitor and 24- (top right) and 48-hour (bottom right) co-treatment with QVD. Each data point represents a biological replicate. Shape of data point corresponds to biological replicate (circle for n=1, square for n=2, and triangle for n=3). Data analyzed using a parametric two-tailed unpaired t-test with Welch's Correction and a 95% confidence level (p-value ≤ 0.05 as the significance threshold). Error bars represent standard error of the mean.

Supplementary Figure 5. MCL-1 inhibition does not affect hNSC proliferation.

(**A**) Immunofluorescent images of proliferating cells (EdU in white) and nuclei (Hoechst in cyan) acquired on SDC microscope at 60X (scale bar = 50µm). Representative images (n=3) of hNSCs treated with

S63845 MCL-1 inhibitor alone and co-treated with QVD after 24-hours (left panel) and 48-hours (right panel). (**B**) Percentage of EdU labeled hNSCs treated with S63845 MCL-1 inhibitor alone and co-treated with QVD after 24-hours (top panel) and 48-hours (bottom panel). Graphs from left to right: cell count of total proliferating cells, early S-phase (whole nuclear labeling), and late S-phase (punctate nuclear labeling) normalized to total cell count; cell count of early S-phase, and late S-phase normalized to total proliferating cells.

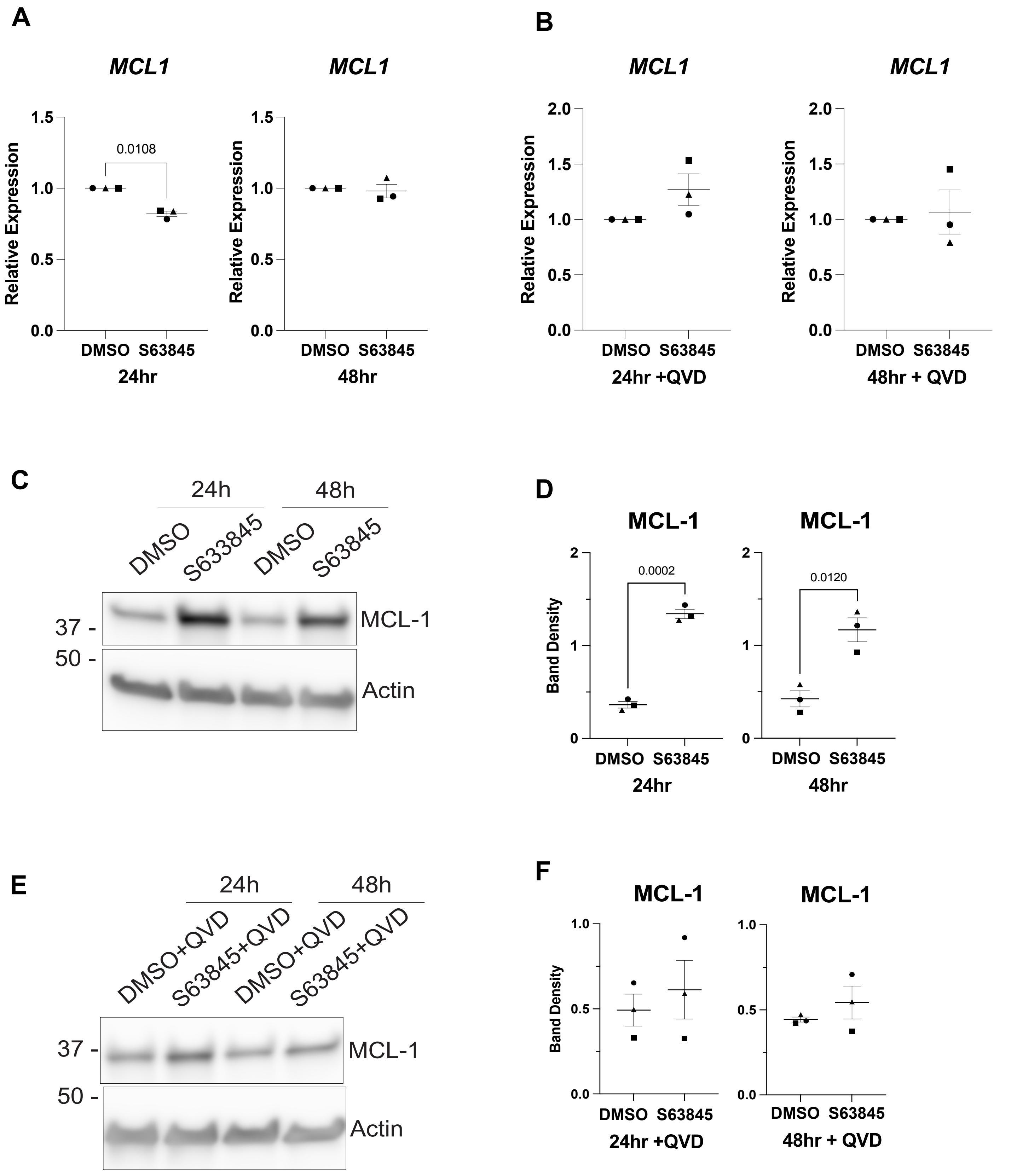
A
MCL1
MCL1
Relative Expression
0.0108
DMSO S63845
24hr
48hr
B
MCL1
MCL1
24hr +QVD
48hr + QVD
C
24h
48h
DMSO
S633845
DMSO
S63845
37 -
50 -
MCL-1
Actin
D
MCL-1
MCL-1
Band Density
0.0002
0.0120
24hr
48hr
E
24h
48h
DMSO+QVD
S63845+QVD
DMSO+QVD
S63845+QVD
37 -
50 -
MCL-1
Actin
F
MCL-1
MCL-1
Band Density
24hr +QVD
48hr + QVD

Supplementary Figure 2

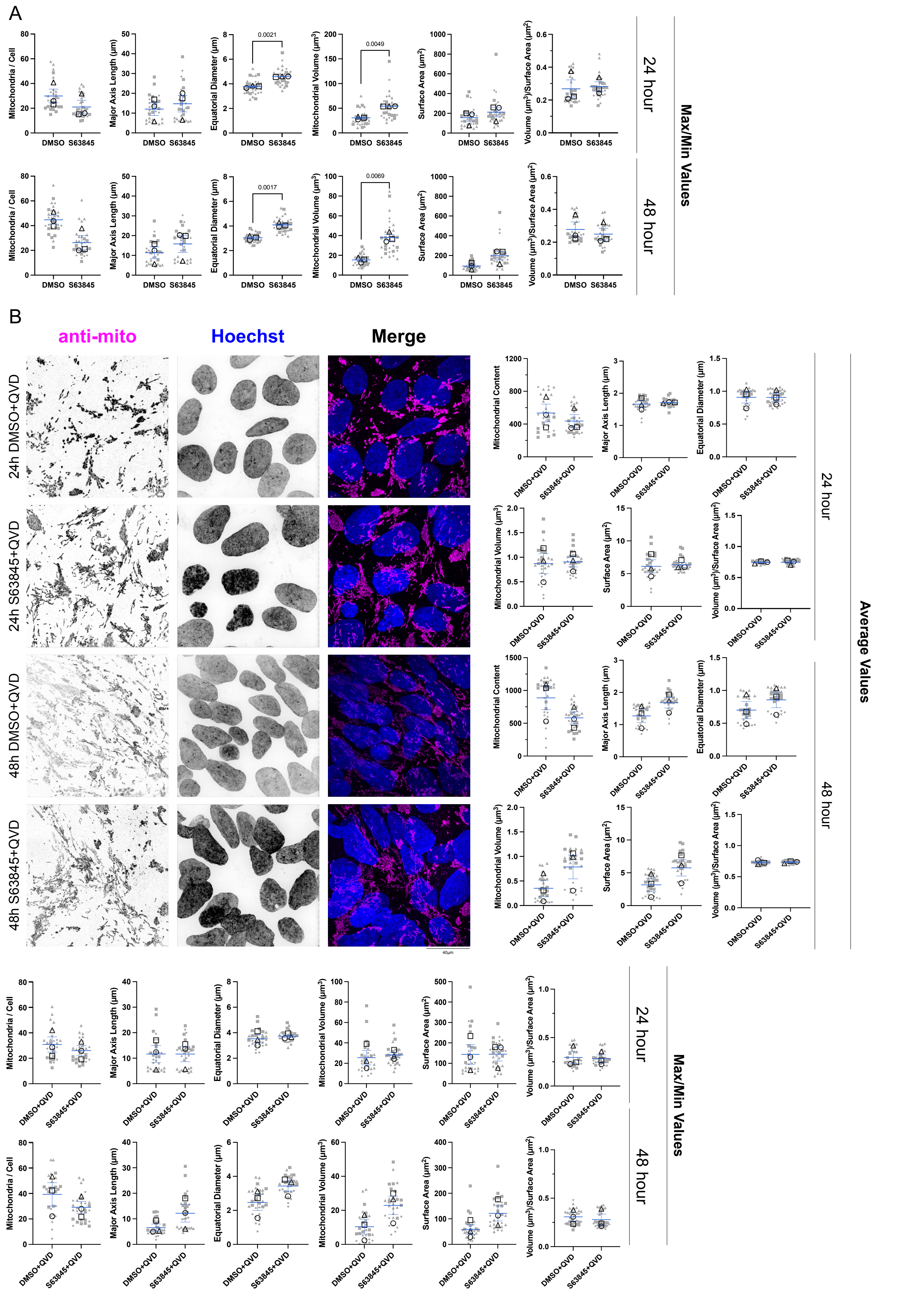

Supplementary Figure 3

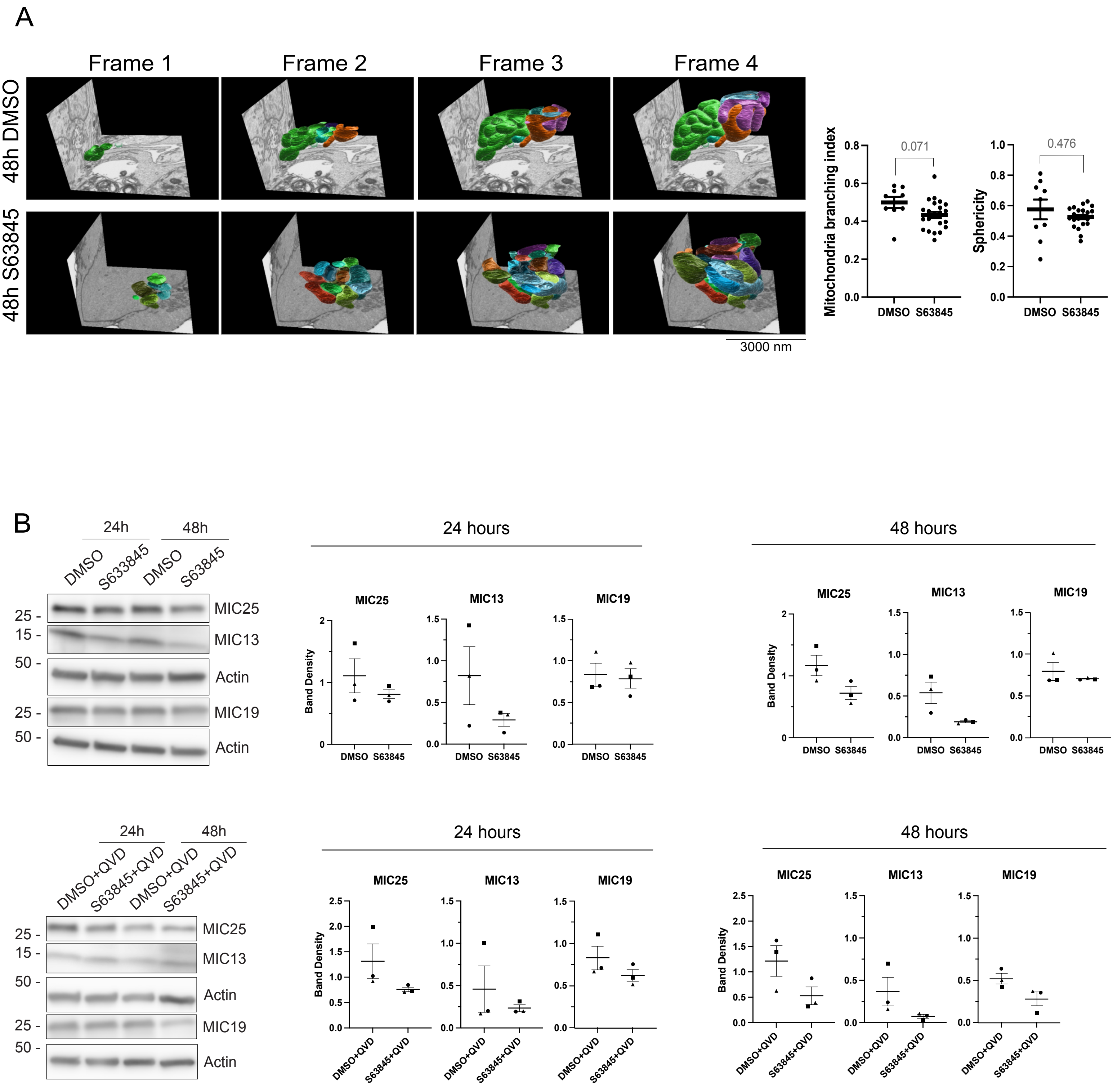

Supplementary Figure 4

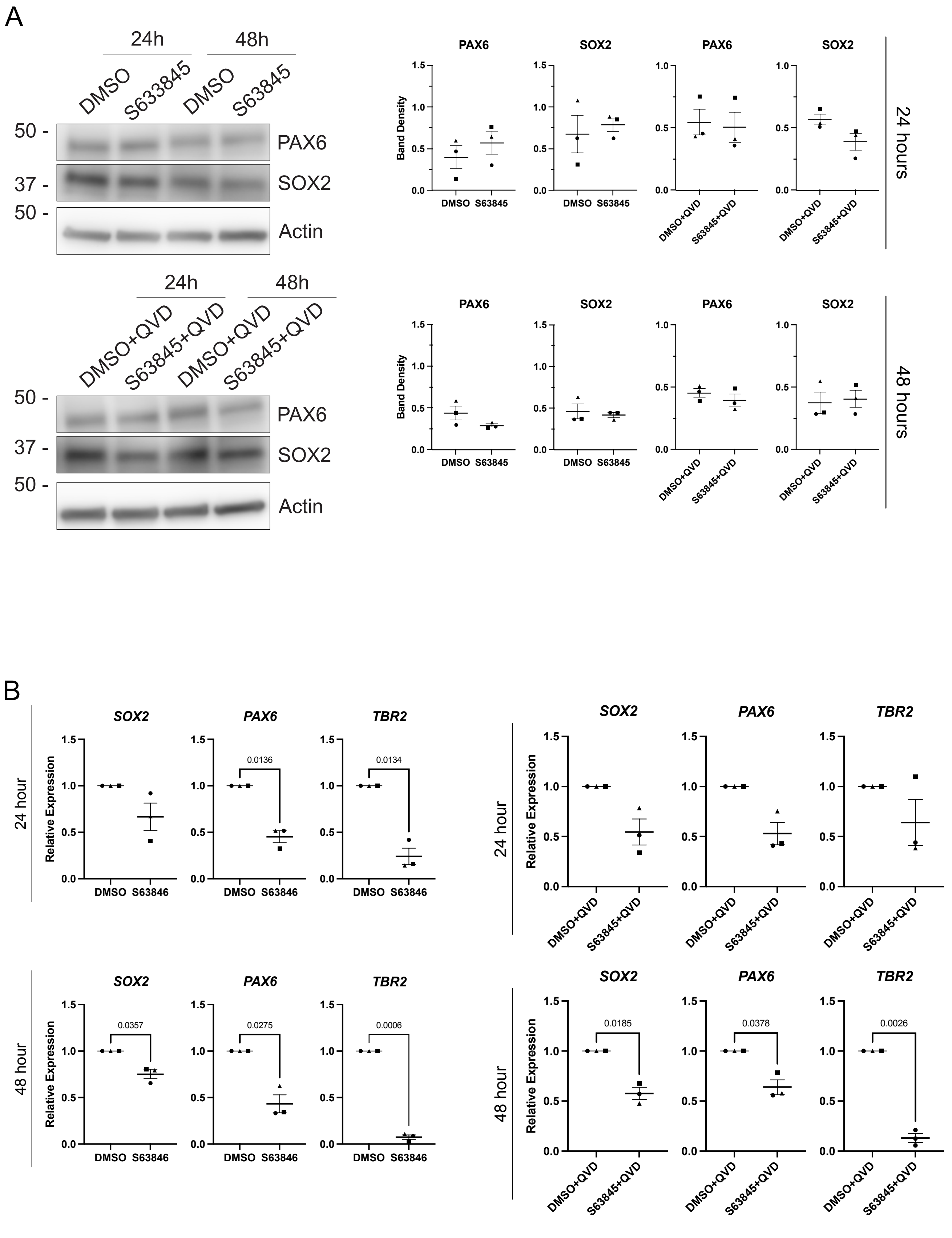

A

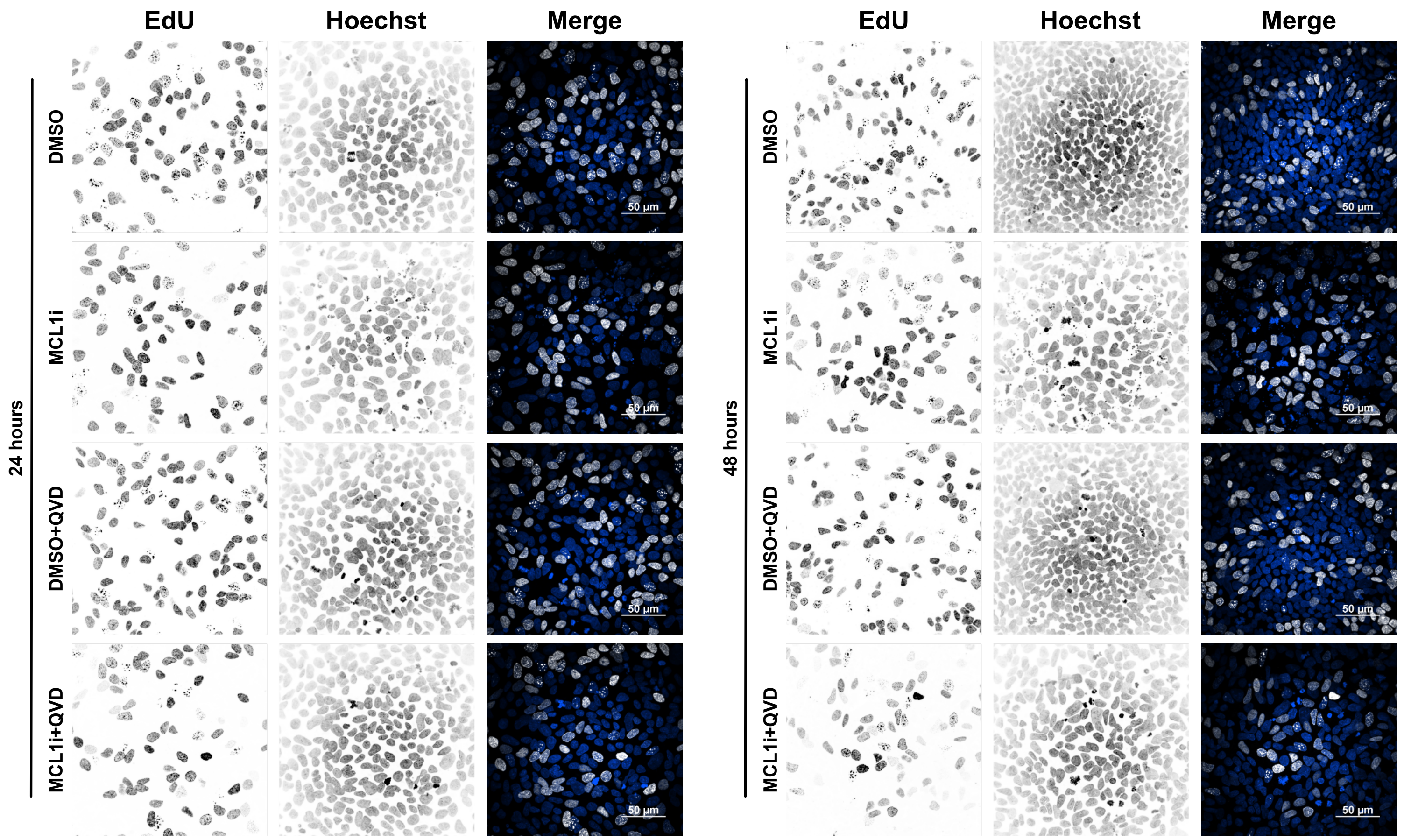
EdU
Hoechst
Merge
24 hours
DMSO
MCL1i
DMSO+QVD
MCL1i+QVD
48 hours
50 µm

B

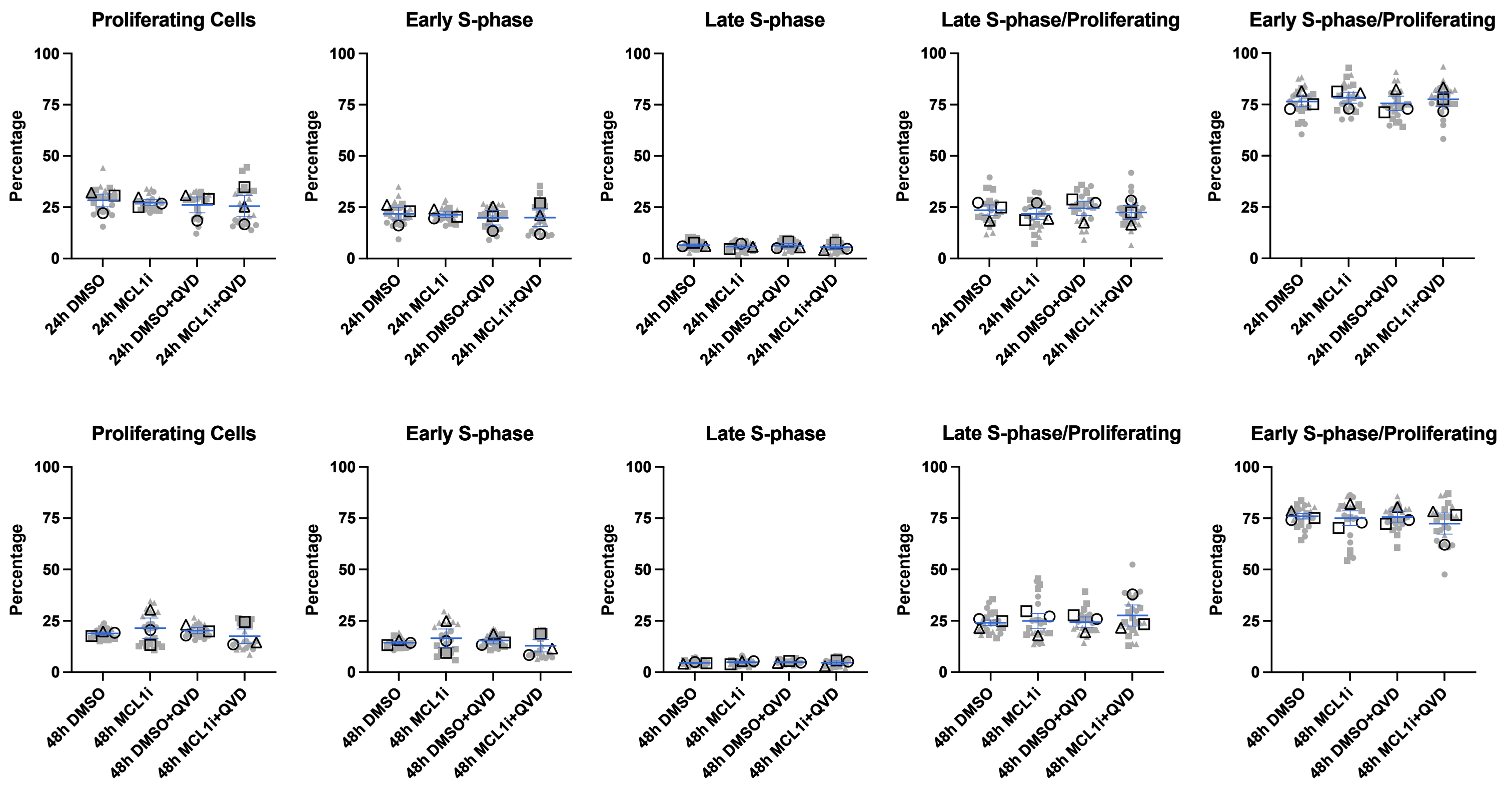
Proliferating Cells
Early S-phase
Late S-phase
Late S-phase/Proliferating
Early S-phase/Proliferating
Percentage
24h DMSO
24h MCL1i
24h DMSO+QVD
24h MCL1i+QVD
48h DMSO
48h MCL1i
48h DMSO+QVD
48h MCL1i+QVD
